# Aortic valve sclerosis is not a benign finding but progressive disease associated with poor cardiovascular outcomes

**DOI:** 10.1186/s44348-024-00037-y

**Published:** 2024-11-26

**Authors:** Jeong Hun Seo, Kwang Jin Chun, Bong-Ki Lee, Byung-Ryul Cho, Dong Ryeol Ryu

**Affiliations:** Division of Cardiology, Department of Internal Medicine, Kangwon National University Hospital, Kangwon National University School of Medicine, Chuncheon, Republic of Korea

**Keywords:** Aortic valve diseases, Echocardiography, Disease progression, Cardiovascular risk

## Abstract

**Background:**

Aortic valve sclerosis (AVS) shares risk factors with atherosclerosis. However, the relationship between AVS progression with cardiovascular (CV) risk has not been researched. This study investigates CV outcomes according to progression of AVS.

**Methods:**

This study included 2,901 patients with AVS (irregular leaflet thickening and peak aortic jet velocity < 2 m/sec) who underwent serial echocardiograms at least 1 year apart during 2011–2020. The primary outcome was defined as CV death, myocardial infarction, stroke, or revascularization.

**Results:**

During a median follow-up period of 3.9 years, 439 of 2,901 AVS patients (15.1%) progressed to mild or greater aortic stenosis. Patients with progression were older and more likely to have atrial fibrillation than those without. In a stepwise regression, age (odds ratio [OR] per 1-year increase, 1.04; 95% confidence interval [CI], 1.01–1.07), peripheral artery disease (OR, 9.07; 95% CI, 3.12–26.4), and left ventricular mass index (OR per 1-g/m^2^ increase, 1.01; 95% CI, 1.00–1.02) were associated with AVS progression. Over a median of 6.3 years, the primary outcome occurred in 858 of 2,901 patients (29.6%). Patients with progression had higher frequency of CV death, myocardial infarction, stroke, or revascularization than those without progression (*P* < 0.0001). In Cox proportional hazards regression, AVS progression (hazard ratio, 1.33; 95% CI, 1.10–1.61) was a significant determinant of CV mortality.

**Conclusions:**

The progression to aortic stenosis in AVS patients is an independent risk factor for CV mortality. These findings suggest that patients with AVS progression may benefit from stricter CV risk monitoring.

**Supplementary Information:**

The online version contains supplementary material available at 10.1186/s44348-024-00037-y.

## Background

Aortic valve sclerosis (AVS) is characterized by focal or diffuse aortic valve thickening without significant hemodynamic obstruction [1]. Although AVS itself may be asymptomatic and not a great medical concern, recent meta-analyses have shown that its presence is associated with higher cardiovascular (CV) events [2, 3]. This may be because AVS shares many risk factors, such as age, sex, smoking, and metabolic syndrome with atherosclerosis [4, 5]. Support for this concept includes the observation that about 50% of those undergoing aortic valve intervention for severe aortic stenosis (AS) have concurrent significant coronary artery disease (CAD) [6].

However, whether AVS should be regarded as a normal degenerative process associated with aging or a serious marker for CV risk is uncertain [7–9]. In addition, unlike AS, AVS has no clear criteria for a monitoring period in the current guidelines. Previous studies have only shown that those with baseline AVS experience more major adverse events compared to the control group [9, 10]. Currently, there is no confirmatory evidence to support routine monitoring for AVS progression.

We hypothesized that progression of AVS to AS would be related to a concerning increase in atherosclerotic CV diseases. Therefore, we sought to investigate CV outcomes according to progression of AVS.

## Methods

### Study population

**Fig. 1:**
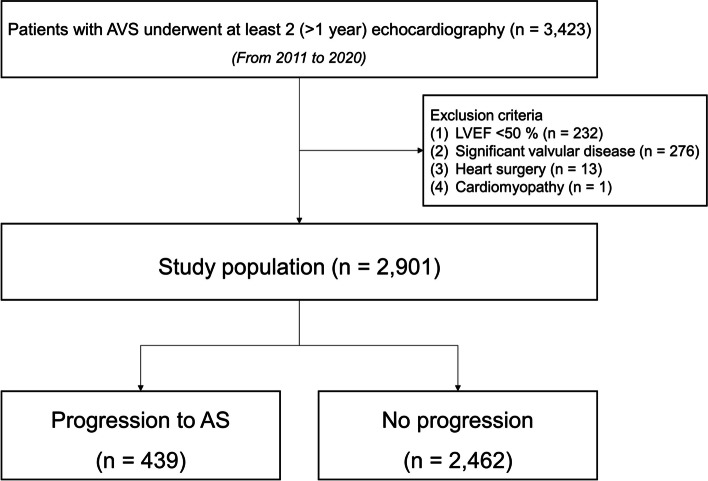
Flowchart of the study. AVS, aortic valve sclerosis; LVEF, left ventricular ejection fraction; AS, aortic stenosis

We retrospectively included 2,901 patients with AVS (irregular leaflet thickening, focally increased echogenicity) revealed by two-dimensional echocardiography and (peak aortic jet velocity [Vmax], < 2 m/sec) by Doppler echocardiography (Fig. S1) and subsequently selected patients who had undergone two or less echocardiographic examinations at least 1 year apart during 2011–2020. Patients with stenosis or regurgitation of at least moderate mitral or tricuspid valve and at least moderate aortic regurgitation, left ventricular dysfunction (left ventricular ejection fraction [LVEF], < 50%), cardiomyopathy or a history of cardiac surgery were excluded. A flowchart is presented on Fig. 1.

### Echocardiography

Comprehensive transthoracic echocardiography was performed using commercially available equipment (Vivid E9, GE Healthcare; Acuson SC2000, Siemens Medical Solutions). Standard M-mode, two-dimensional, and color Doppler imaging were performed in parasternal, suprasternal, substernal, and apical views with positional adjustment of the patient. At the time of follow-up echocardiographic examinations, AS was classified as mild (aortic valve area [AVA], 1.5–2.0 cm^2^; Vmax, 2.0–3.0 m/sec), moderate (AVA, 1.0–1.5 cm^2^; Vmax, 3.0–4.0 m/sec), or severe (AVA, < 1.0 cm^2^, Vmax, > 4.0 m/sec). Anatomic measurements were performed according to the current guidelines [11].

### Measurement of covariables

At the time of baseline echocardiographic examinations, relevant CV risk factors were assessed by a complete review of patient medical records (smoking, body mass index, blood pressure, medication, or laboratories). Dyslipidemia was defined as a total cholesterol > 200 mg/dL and/or use of lipid-lowering therapy; diabetes mellitus was defined as a fasting plasma glucose > 126 mg/dL and/or use of antidiabetic medication; hypertension was defined as a systolic blood pressure > 140 mmHg and/or use of antihypertensive medication; CAD was defined as previously documented myocardial infarction (MI) and/or coronary artery stenosis with a lumen diameter > 50% on angiography; peripheral artery disease (PAD) was defined as an ankle-brachial index less than 0.9 and/or peripheral artery stenosis > 50% on angiography. Cerebrovascular accident (CVA) was defined as the presence of neurologic symptoms and/or abnormal lesions on brain imaging, and atrial fibrillation (AF) was defined as a documented irregular rhythm on electrocardiogram regardless of duration.

### CV outcomes

The primary outcome was defined as the composite of CV death, MI, or revascularization or as stroke. The secondary outcome was each component of CV death, MI, or revascularization and stroke. The count of CV events was measured at least 3 months after the initial date.

### Statistical analysis

Continuous variables were tested for normality using the Shapiro–Wilk test. Results were expressed as mean ± standard deviation or median (interquartile range) and compared with Student t test or the Wilcoxon rank sum test between patients with progression to AS versus the group with no progression. Categorical variables are presented as percentages and were compared with the chi-square test or Fisher exact test, as appropriate. Backward stepwise regression was performed to assess the factors associated with progression to AS in patients with AVS after adjusting for clinically relevant variables at baseline: age, sex, body mass index, smoking, hypertension, diabetes, dyslipidemia, CAD, PAD, AF, hemoglobin, high-sensitive C-reactive protein (hsCRP), uric acid, LVEF, left ventricular mass index (LVMI), left atrial volume index (LAVI), early mitral inflow velocity to early diastolic mitral annular velocity ratio (E/e′), right ventricular systolic pressure (RVSP), and Vmax. The cumulative incidence of CV events was evaluated by Kaplan–Meier analyses and the level of significance was assessed with the log-rank test. A regression analysis using Cox proportional hazards modeling was performed to identify independent predictors of CV outcomes. Missing data percentages were 8.5% for hsCRP level, 0.3% for uric acid, 3.8% for LVMI, 1.4% for LAVI, 1.6% for E/e′, and 3.8% for RVSP. There were no missing data for age, sex, body mass index, smoking, hypertension, diabetes, dyslipidemia, CAD, PAD, AF, hemoglobin, LVEF, and Vmax. We used Little test for missing completely at random to validate whether data were missing. All statistical tests were two-tailed and *P* < 0.05 was considered statistically significant. Statistical analyses were performed using the R ver. 4.2.2 (R Foundation for Statistical Computing) and IBM SPSS ver. 25.0 (IBM Corp).

## Results

### Baseline characteristics

Of the 2,901 AVS patients with two echo examinations, 439 (15.1%) progressed to mild or greater AS during a median follow-up of 3.9 years (IQR, 2.1–6.1 years; progression group, 4.6 years [IQR, 2.5–6.7 years] vs. no progression group, 3.8 years [IQR, 2.1–6.0 years]). Baseline characteristics are listed in Table 1, stratified by progression to AS. Patients with progression were older, less often male, more often PAD and AF, had higher blood urea nitrogen and uric acid levels and lower hemoglobin, and used P2Y12 inhibitors, loop diuretics, and statins more frequently than those without progression. In echocardiographic parameters, patients with progression had higher LVMI, LAVI, late diastolic mitral inflow velocity, E/e′, and Vmax (Table 2). The echocardiographic parameters at follow-up are listed in Table S1.

**Table 1:** Baseline characteristics

Characteristic	Overall (*n* = 2,901)	Progression to aortic stenosis		
		Yes (*n* = 439)	No (*n* = 2,462)	*P*-value
Clinical data				
Age (yr)	70.6 ± 10.6	74.0 ± 9.0	70.0 ± 10.7	< 0.001^*^
Male sex	1,212 (41.8)	153 (34.9)	1,059 (43.0)	0.002^*^
Body mass index (kg/m^2^)	25.1 ± 4.1	24.8 ± 4.2	25.1 ± 4.1	0.130
Systolic blood pressure (mmHg)	129.6 ± 18.7	131.0 ± 19.4	129.4 ± 18.6	0.097
Diastolic blood pressure (mmHg)	76.7 ± 11.1	76.5 ± 11.1	76.7 ± 11.0	0.727
Ever smoking	139 (4.8)	16 (3.6)	123 (5.0)	0.271
Hypertension	2,198 (75.8)	343 (78.1)	1,855 (75.3)	0.232
Diabetes mellitus	980 (33.8)	145 (33.0)	835 (33.9)	0.759
Dyslipidemia	1,768 (60.9)	282 (64.2)	1,486 (60.4)	0.138
Prior coronary artery disease	648 (22.3)	104 (23.7)	544 (22.1)	0.499
Prior cerebrovascular accident	698 (24.1)	114 (26.0)	584 (23.7)	0.340
Peripheral artery disease	33 (1.1)	11 (2.5)	22 (0.9)	0.007^*^
Atrial fibrillation	429 (14.8)	85 (19.4)	344 (14.0)	0.004^*^
Medication				
Aspirin	1,627 (56.1)	262 (59.7)	1,365 (55.4)	0.110
P2Y12 inhibitor	1,055 (36.4)	179 (40.8)	876 (35.6)	0.042^*^
Vitamin K antagonist	159 (5.5)	33 (7.5)	126 (5.1)	0.055
ACE inhibitor	60 (2.1)	14 (3.2)	46 (1.9)	0.108
Angiotensin receptor blocker	1,542 (53.2)	236 (53.8)	1,306 (53.0)	0.823
β-blocker	922 (31.8)	151 (34.4)	771 (31.3)	0.222
Calcium channel blocker	1,019 (35.1)	169 (38.5)	850 (34.5)	0.121
Loop diuretic	643 (22.2)	122 (27.8)	521 (21.2)	0.003^*^
Spironolactone	249 (8.6)	38 (8.7)	211 (8.6)	> 0.999
Thiazide-like diuretic	419 (14.4)	68 (15.5)	351 (14.3)	0.546
Statin	1,689 (58.2)	277 (63.1)	1,412 (57.4)	0.028^*^
Laboratory data				
Hemoglobin (g/dL)	12.6 ± 2.0	12.2 ± 2.0	12.6 ± 2.0	< 0.001^*^
hsCRP (mg/dL)	0.2 (0.1–1.5)	0.2 (0.1–1.7)	0.2 (0.1–1.5)	0.393
Blood urea nitrogen (mg/dL)	18.3 ± 10.6	20.2 ± 11.6	17.9 ± 10.4	< 0.001^*^
Creatinine (mg/dL)	0.8 (0.6–1.0)	0.8 (0.7–1.1)	0.8 (0.6–1.0)	0.070
Uric acid (mg/dL)	5.2 ± 1.8	5.4 ± 2.0	5.2 ± 1.8	0.018^*^
Glucose (mg/dL)	132.4 ± 58.9	129.7 ± 57.6	132.8 ± 59.1	0.310
Calcium (mg/dL)	9.0 ± 0.6	9.0 ± 0.6	9.0 ± 0.6	0.612
HbA1c (%)	6.6 ± 1.4	6.6 ± 1.4	6.5 ± 1.4	0.507
Total cholesterol (mg/dL)	164.6 ± 42.2	162.3 ± 41.8	165.0 ± 42.3	0.216
LDL cholesterol (mg/dL)	97.5 ± 37.0	95.4 ± 38.6	97.8 ± 36.7	0.252
HDL cholesterol (mg/dL)	44.9 ± 13.7	45.4 ± 13.3	44.9 ± 13.8	0.486
Triglyceride (mg/dL)	141.0 ± 90.2	137.1 ± 90.1	141.7 ± 90.2	0.369

Values are presented as mean ± standard deviation, number (%), or median (interquartile range)

*ACE* angiotensin-converting enzyme, *hsCRP* high-sensitivity C-reactive protein, *HbA1c* hemoglobin A1c, *LDL* low-density lipoprotein, *HDL* high-density lipoprotein

^*^P < 0.05 (statistically significant)

**Table 2:** Echocardiographic parameters

Parameter	Overall (*n* = 2,901)	Progression to AS		
		Yes (*n* = 439)	No (*n* = 2,462)	*P*-value
LV end-diastolic dimension (mm)	47.1 ± 4.7	46.9 ± 4.8	47.2 ± 4.7	0.201
LV end-systolic dimension (mm)	29.6 ± 4.1	29.3 ± 4.1	29.6 ± 4.1	0.176
LVEF (%)	65.8 ± 5.8	65.8 ± 5.8	65.8 ± 5.8	0.929
LVMI (g/m^2^)	94.6 ± 22.9	97.3 ± 22.3	94.2 ± 23.0	0.030^*^
LAVI (mL/m^2^)	38.7 ± 14.7	40.3 ± 14.0	38.4 ± 14.9	0.013^*^
E (m/sec)	0.6 ± 0.2	0.6 ± 0.2	0.6 ± 0.2	0.428
A (m/sec)	0.9 ± 0.2	0.9 ± 0.2	0.8 ± 0.2	< 0.001^*^
E/e′	11.7 ± 4.6	12.3 ± 4.7	11.7 ± 4.6	0.009^*^
RVSP (mmHg)	27.8 ± 8.1	28.2 ± 7.9	27.8 ± 8.2	0.273
Peak aortic jet velocity (m/sec)	1.7 ± 0.2	1.8 ± 0.2	1.7 ± 0.1	< 0.001^*^

Values are presented as mean ± standard deviation

*LV* left ventricle, *LVEF* left ventricular ejection fraction, *LVMI* left ventricular mass index, *LAVI* left atrial volume index, *E* early diastolic mitral inflow velocity, *A* late diastolic mitral inflow velocity, *E/e*′ early mitral inflow velocity to early diastolic mitral annular velocity ratio, *RVSP* right ventricular systolic pressure

^*^P < 0.05 (statistically significant)

### Factors associated with AVS progression

Among 439 AVS patients with progression, most progressed to mild AS, while only 21 progressed to moderate and severe AS (Fig. S2). After adjustment for clinically relevant variables by backward elimination, age (odds ratio [OR] per 1-year increase, 1.04; 95% confidence interval [CI], 1.01–1.07), PAD (OR, 9.07; 95% CI, 3.12–26.4), and LVMI (OR per 1-g/m^2^ increase, 1.01; 95% CI, 1.00–1.02) were significantly associated with progression to AS in AVS patients (Fig. S3).

### CV outcomes according to AVS progression

**Fig. 2:**
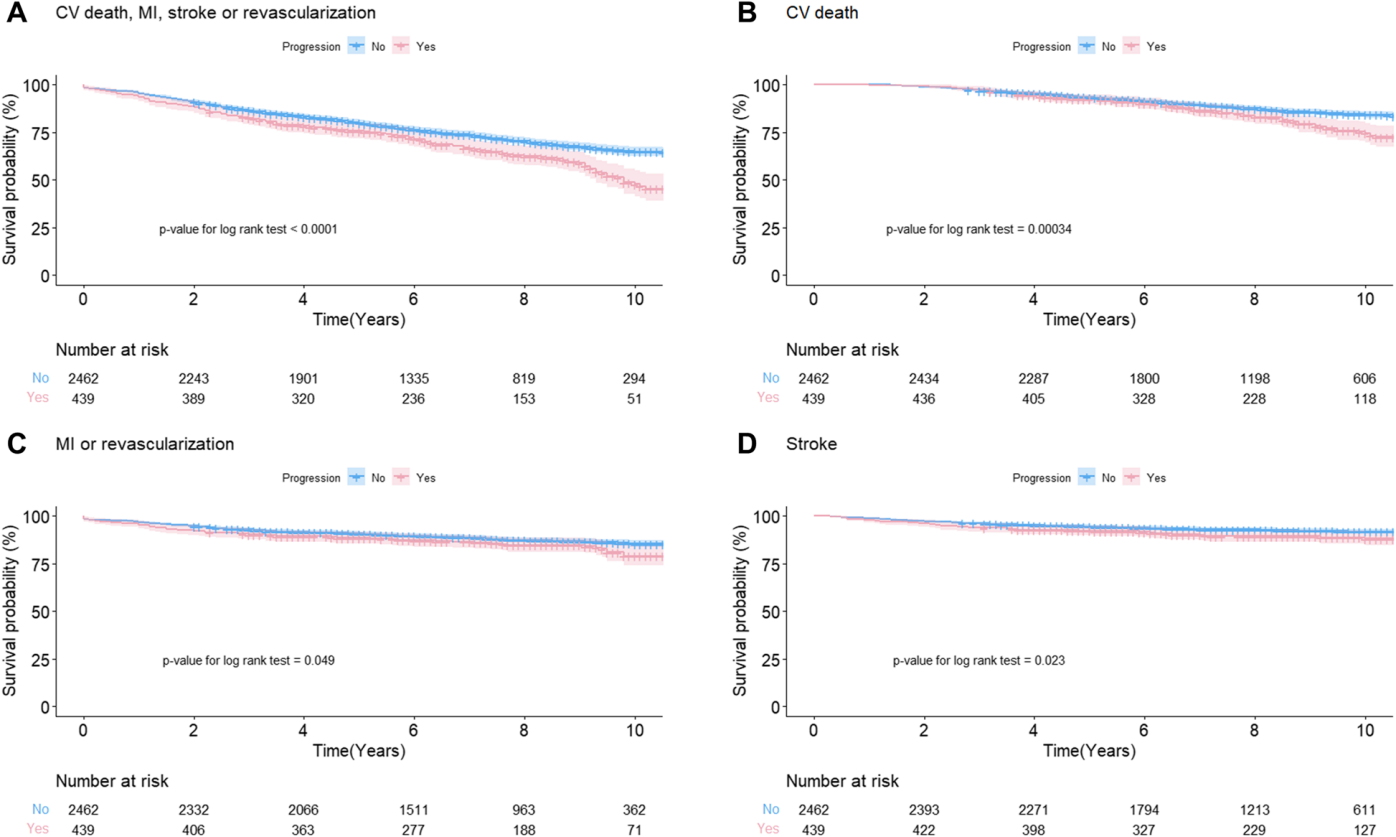
Kaplan–Meier curves between aortic valve sclerosis patients with and without progression to aortic stenosis. **A** Cardiovascular (CV) death, myocardial infarction (MI), stroke, or revascularization. **B** CV death. **C** MI or revascularization. **D** Stroke

**Fig. 3:**
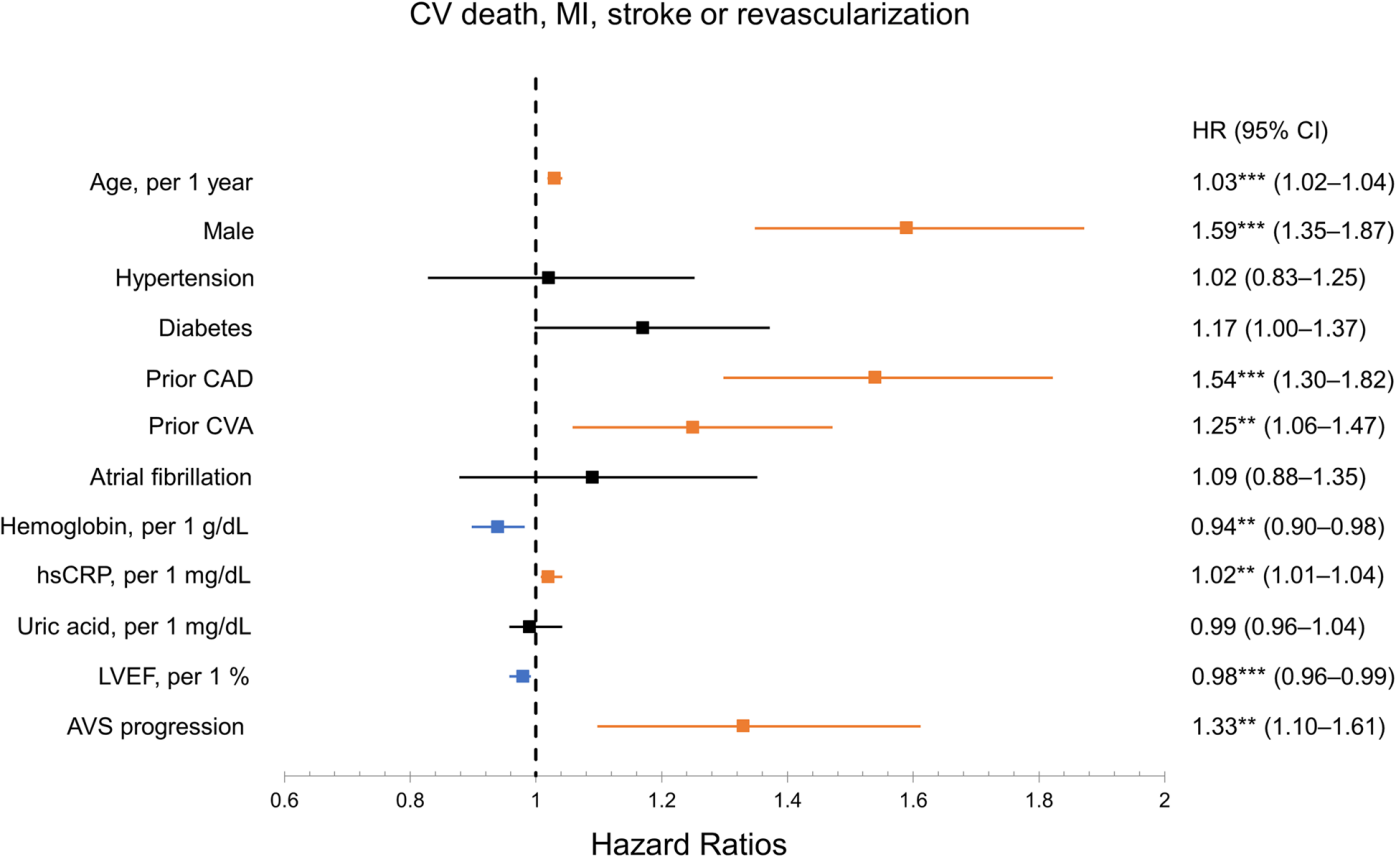
Forest plot for Cox proportional hazard models shows multivariate Cox regression analysis of the effects of parameters on the primary outcome. CV, cardiovascular; MI, myocardial infarction; HR, hazard ratio; CI, confidence interval; CAD, coronary artery disease; CVA, cerebrovascular accident; hsCRP, high-sensitive C-reactive protein; LVEF, left ventricular ejection fraction; AVS, aortic valve sclerosis. ^*^P < 0.05, ^**^P < 0.005, ^***^P < 0.001

During a median follow-up of 6.3 years (IQR, 4.1–8.8 years), 858 patients (29.6%) experienced the primary outcome, and AVS patients with progression had more frequent CV events (Table 3). There was a statistically significant increased risk of CV death, MI, stroke, or revascularization in AVS patients with progression (*P* < 0.0001) (Fig. 2A). Each CV death **(**Fig. 2B**)**, MI or revascularization **(**Fig. 2C**)**, and stroke **(**Fig. 2D**)** showed consistent results. In a Cox proportional hazards regression model, age (hazard ratio [HR] per 1-year increase, 1.03; 95% CI, 1.02–1.04), male sex (HR, 1.59; 95% CI, 1.35–1.87), prior CAD (HR, 1.54; 95% CI, 1.30–1.82), prior CVA (HR, 1.25; 95% CI, 1.06–1.47), hsCRP (HR per 1-mg/dL increase, 1.02; 95% CI, 1.01–1.04), and AVS progression (HR, 1.33; 95% CI, 1.10–1.61) were significant determinants of CV mortality (Fig. 3). Hemoglobin (HR per 1-g/dL increase, 0.94; 95% CI, 0.90–0.98) and LVEF (HR per 1%-increase, 0.98; 95% CI, 0.96–0.99) ameliorated CV risk.

**Table 3:** Cardiovascular outcomes

Outcome	Overall (*n* = 2,901)	Progression to aortic stenosis		
		Yes (*n* = 439)	No (*n* = 2,462)	*P*-value
Primary outcome	858 (29.6)	172 (39.2)	686 (27.9)	< 0.001^*^
Cardiovascular death	398 (13.7)	87 (19.8)	311 (12.6)	< 0.001^*^
Myocardial infarction or revascularization	359 (12.4)	68 (15.5)	291 (11.8)	0.031^*^
Stroke	224 (7.7)	46 (10.5)	178 (7.2)	0.019^*^

Values are presented as number (%)

^*^P < 0.05 (statistically significant)

### Sensitivity analysis

A total of 1,220 patients had CAD and CVA at baseline echocardiography. Aside from these patients, AVS progression was a significant factor in CV mortality (HR, 1.32; 95% CI, 1.03–1.71). There was a significant difference in CV mortality according to progression of AVS (Fig. S4). Stroke, MI, and revascularization were not significant (data not shown).

## Discussion

The main findings of this study are as follows: (1) during a median follow-up period of 3.9 years, 439 of 2,901 AVS patients (15.1%) progressed to mild or greater AS, and progression was associated with age, PAD, or LVMI; (2) during a median follow-up period of 6.3 years, 858 of 2,901 AVS patients (29.6%) experienced CV events, and patients with progression had more frequent CV death, MI, stroke, or revascularization than those without progression; and (3) AVS progression was a significant determinant of CV mortality regardless of prior CAD or CVA.

### Progression of AVS to AS

There have been few prospective studies following rates of hemodynamic progression spanning the disease spectrum of AVS to AS. In two population studies, 1.8% to 1.9% of subjects with AVS progressed to clinical AS each year [12, 13]. Our study found an annual progression rate of 3.7%. The reason for the higher rate in our cohort is thought to be due to older age, more frequent comorbidities, or different criteria for AS (e.g., at least moderate). The rate is low in those who progressed to moderate or severe AS (only 5% over 4 years) in this study. Previous studies showed that only 1% of those with normal valves developed AS over 5 years compared with 9% of those with AVS [13] and none of those with normal valves at baseline developed moderate or severe AS [12]. These findings indicate that AVS is a separate disease process and a necessary step in progression to AS.

Some studies have assessed the relationship between AVS progression and atherosclerotic CV risk factors [9, 10, 14]. In our study, risk factors for AVS progression are age, PAD, and LVMI. Age and PAD are associated with atherosclerosis and LVMI is also related to atherosclerosis in terms of left ventricular hypertrophy caused by hypertension. Previous studies showed that clinical factors such as hypertension, diabetes, smoking, and dyslipidemia can predict the incidence of AVS but did not predict hemodynamic progression of AVS [5, 15]. In two studies, the extent of baseline calcification and male sex were associated with a higher rate of progression [12, 16]. Therefore, atherosclerosis seems to be a necessary but not sufficient link for progression of AVS to AS.

### CV outcomes according to AVS progression

Previous studies have demonstrated that the presence of AVS is associated with a relevant increase in CV mortality, MI, and heart failure, even in the absence of hemodynamically significant AS [2, 3, 17]. We confirmed that AVS patients who proceed to AS higher CV death, MI, stroke or revascularization than those without progression.

The mechanism of the association of CV risk with AVS progression is not entirely clear. One hypothesis to explain adverse outcomes is that disease progression in the valve leaflets may lead to increased leaflet stiffness with valve obstruction (e.g., AS). In a study of more than 2,000 patients with AVS, progression to AS occurred in 16% and most developed only mild stenosis [18]. Our study shows similar patterns. A prospective study revealed that baseline Vmax, progression rate, and functional status were independent predictors for mortality in patients with Vmax above 2.5 m/sec [19]. Another study found that the presence of moderate or severe valvular calcification, together with a rapid increase in Vmax was a poor prognostic factor in those with severe AS [20]. The results of previous studies are difficult to apply directly to our study because the target group and the progression rate are different.

Another hypothesis is that AVS and atherosclerosis are the result of a common underlying pathophysiologic mechanism such as inflammation or endothelial dysfunction [21]. Convincing evidence indicates that the presence of AVS in comparison to normal controls is significantly associated with subclinical carotid atherosclerosis, endothelial dysfunction and, in turn, an increased CV risk [22–24]. In addition, our study indicates that older age, male sex, higher hsCRP level, and AVS progression are significant determinants of CV mortality. Rather than adverse CV outcomes due to a primary valvular disorder, it has been proposed that AVS progression may represent a surrogate marker either for underlying atherosclerosis or systemic process, such as inflammation [25, 26].

### Monitoring AVS progression for CV risk

Currently, routine screening for AVS is not recommended because it has slower progression than AS. In our study, those who proceeded from AVS to AS for about 4 years experienced more frequent adverse CV events in a median of 6.3 years. This finding suggests that even AVS without hemodynamic significance requires follow-up. Enrolled patients had high CV risks such as prior CAD or stroke, AF, hypertension or dyslipidemia. Thus, the addition of echocardiography could be helpful in the evaluation of patients with high CV risk, because pathologic processes in the CV system may be identified more easily in the aortic valve.

### Limitations

Some potential limitations of our study need to be discussed. First, the retrospective nature of the study does not exclude other potential confounding variables not included in the analysis that could have affected the results. Second, only patients who underwent follow-up echocardiography were included in this study; therefore, selection bias might have affected the results. Third, echocardiography is less sensitive to detecting aortic valve calcification than computed tomography, so it may miss the initial change in calcific aortic valve disease. However, echocardiography was suitable for monitoring the hemodynamic progression of aortic valve disease in the current study. Fourth, interobserver variability was possible because the definition of AVS was subjective. Finally, this study limits the participants to a single center and a single ethnicity. Hence, our findings should be expanded and further verified in well-controlled prospective studies.

## Conclusions

In our AVS cohort, 15.1% of patients progressed to mild or greater AS over 3.9 years and risk factors for progression were age, PAD, or LVMI. In addition, 29.6% of AVS patients experienced CV death, MI, stroke, or revascularization in 6.3 years, and progression to AS is an independent risk factor for CV mortality. These findings suggest that patients with AVS progression may benefit from stricter CV risk monitoring.

## Supplementary Information

Additional file 1: Fig. S1. Presence of aortic valve calcification without significant hemodynamic compromise, typically a peak velocity < 2 m/sec in aortic valve sclerosis. Fig. S2. The proportions of aortic disease grades at follow-up. Fig. S3. Forest plot of multivariate logistic regression for progression in patients with aortic valve sclerosis (AVS). Fig. S4. Kaplan–Meier curves between aortic valve sclerosis patients (no prior coronary artery disease or cerebrovascular accident) who progress to aortic stenosis and those who do not.

Additional file 2: Table S1. Follow-up echocardiographic parameters.

## Data Availability

The data underlying this article will be shared on reasonable request to the corresponding author.

## Abbreviations

AF: Atrial fibrillation
AS: Aortic stenosis
AVA: Aortic valve area
AVS: Aortic valve sclerosis
CAD: Coronary artery disease
CI: Confidence interval
CV: Cardiovascular
CVA: Cerebrovascular accident
E/e': Early mitral inflow velocity to early diastolic mitral annular velocity ratio
HR: Hazard ratio
hsCRP: High-sensitive C-reactive protein
LAVI: Left atrial volume index
LVEF: Left ventricular ejection fraction
LVMI: Left ventricular mass index
MI: Myocardial infarction
OR: Odds ratio
PAD: Peripheral artery disease
RVSP: Right ventricular systolic pressure
Vmax: Peak aortic jet velocity
