# Supplementary material for: Aortic valve sclerosis is not a benign finding but progressive disease associated with poor cardiovascular outcomes
Source: J Cardiovasc Imaging. 2024 Nov 26;32:39. doi: 10.1186/s44348-024-00037-y (PMC11590455; doi:10.1186/s44348-024-00037-y)
Supplement: Supplementary file 1 — Additional file 1: Fig. S1. Presence of aortic valve calcification without significant hemodynamic compromise, typically a peak velocity < 2 m/sec in aortic valve sclerosis. Fig. S2. The proportions of aortic disease grades at follow-up. Fig. S3. Forest plot of multivariate logistic regression for progression in patients with aortic valve sclerosis (AVS). Fig. S4. Kaplan–Meier curves between aortic valve sclerosis patients (no prior coronary artery disease or cerebrovascular accident) who progress to aortic stenosis and those who do not. [file 44348_2024_37_MOESM1_ESM.docx]

**Additional file 1.** Supplementary Figures


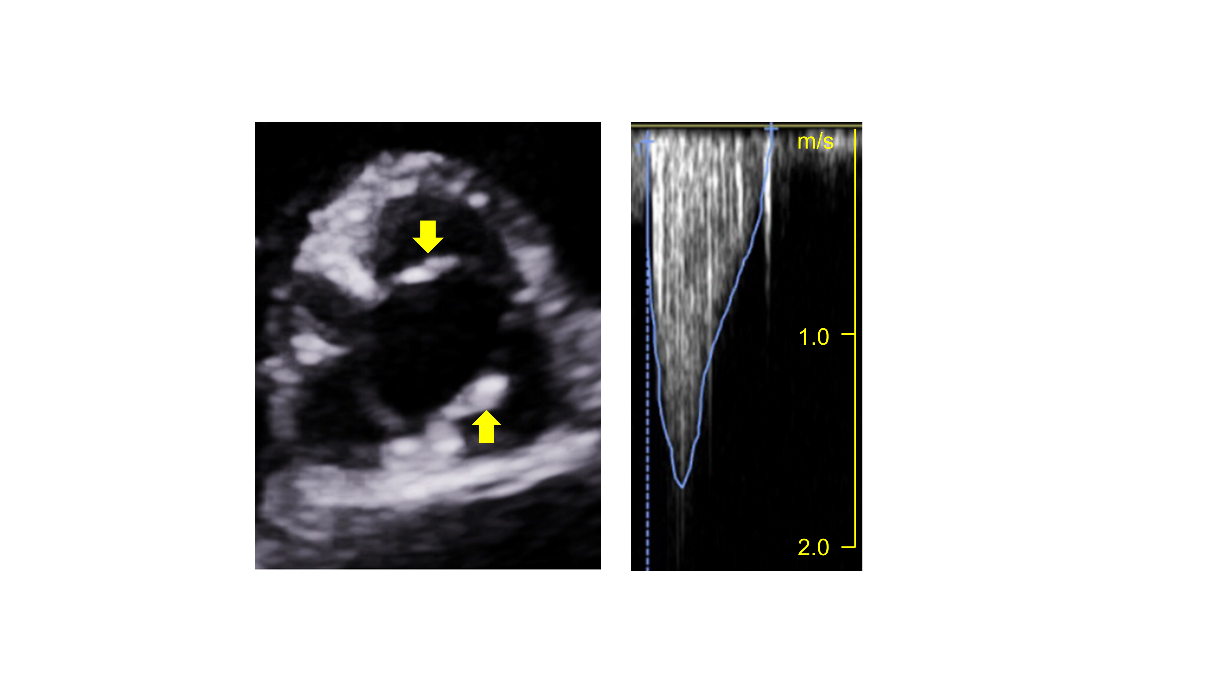


**Fig. S1.** Presence of aortic valve calcification without significant hemodynamic compromise, typically a peak velocity <2 m/s in AVS. Arrows indicate areas of valvular sclerosis.


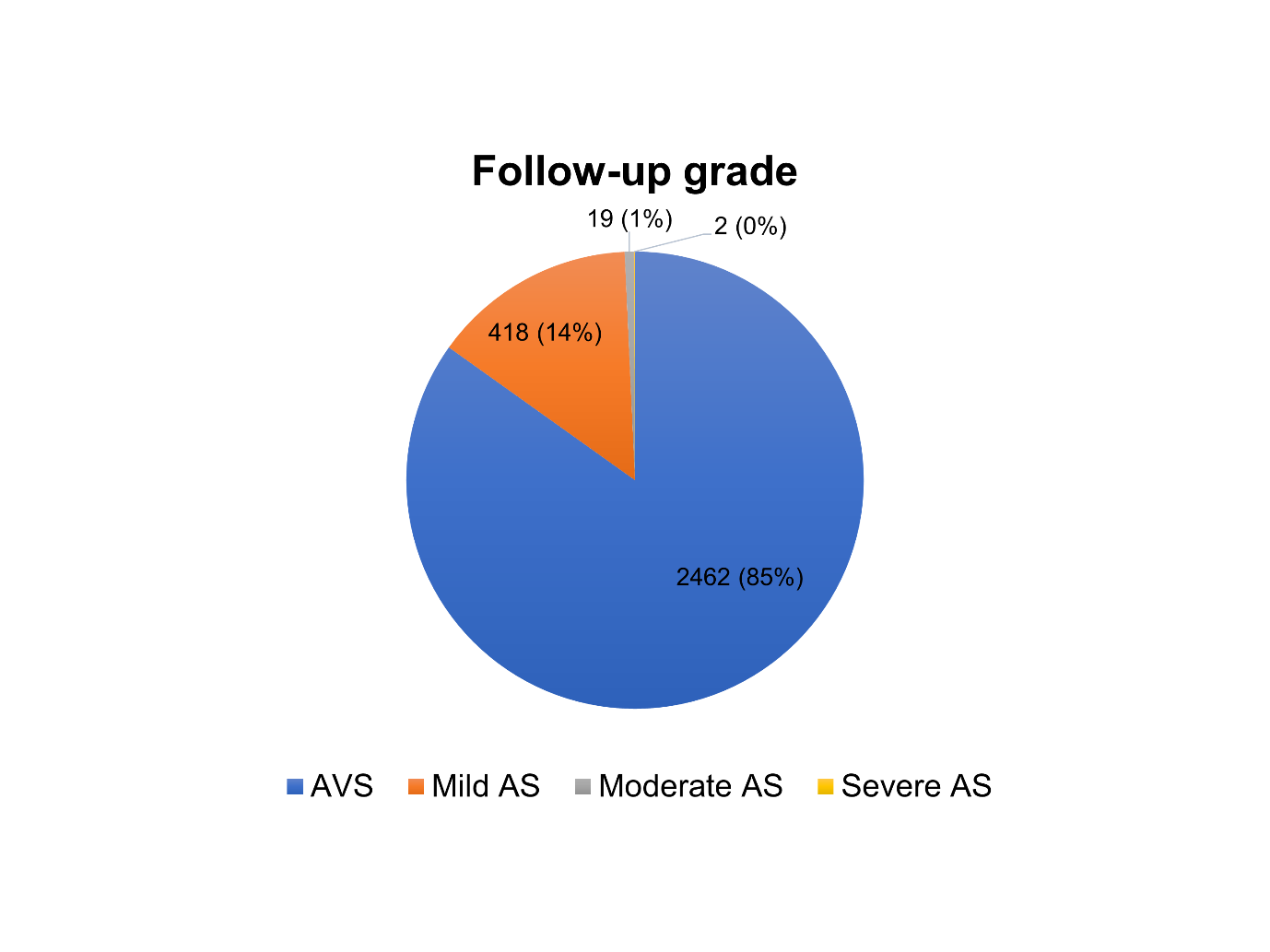


**Fig. S2.** The proportions of aortic disease grades at follow-up. AVS, aortic valve sclerosis; AS, aortic stenosis.


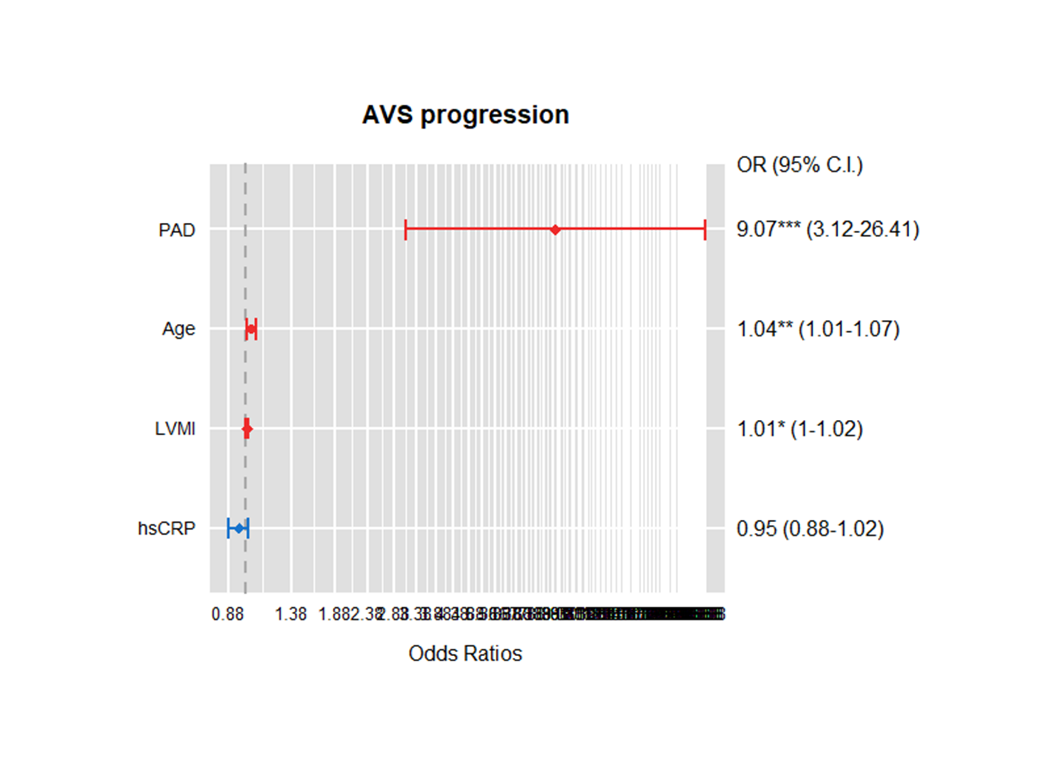


**Fig. S3.** Forest plot of multivariate logistic regression for progression in patients with aortic valve sclerosis (AVS). OR, odds ratio; CI, confidence interval; PAD, peripheral artery disease; LVMI, left ventricular mass index; hsCRP, high-sensitive C-reactive protein. ^*^P<0.05, ^**^P<0.005, ^***^P<0.001.


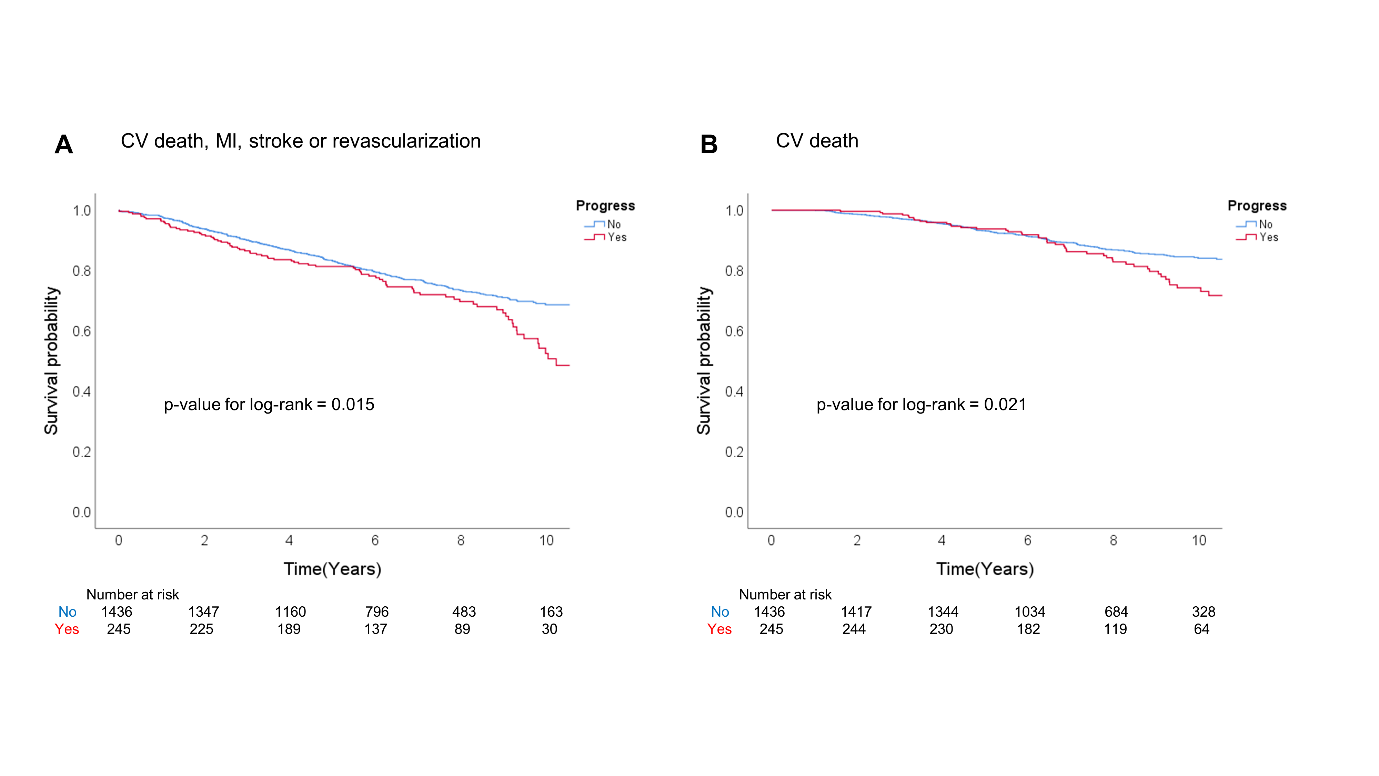


**Fig. S4.** Kaplan-Meier curves between aortic valve sclerosis patients (no prior coronary artery disease or cerebrovascular accident) who progress to aortic stenosis and those who do not. (A) Cardiovascular (CV) death, myocardial infarction (MI), stroke, or revascularization. (B) CV death.
