# Supplementary material for: Aortic valve sclerosis is not a benign finding but progressive disease associated with poor cardiovascular outcomes
Source: J Cardiovasc Imaging. 2024 Nov 26;32:39. doi: 10.1186/s44348-024-00037-y (PMC11590455; doi:10.1186/s44348-024-00037-y)
Supplement: Supplementary file 2 — Additional file 2: Table S1. Follow-up echocardiographic parameters. [file 44348_2024_37_MOESM2_ESM.docx]

| **Supplementary Table 1. Follow-Up echocardiographic parameters** | | | | |
| --- | --- | --- | --- | --- |
|  | Overall  (n=2901) | Progression to AS  (n=439) | No progression  (n=2462) | ***p value |
| Left ventricular end-diastolic dimension (mm) | 46.7 ± 5.4 | 46.8 ± 5.6 | 46.7 ± 5.4 | 0.762 |
| Left ventricular end-systolic dimension (mm) | 30.3 ± 5.0 | 30.2 ± 4.9 | 30.3 ± 5.0 | 0.613 |
| Left ventricular ejection fraction (%) | 62.8 ± 7.1 | 63.3 ± 6.9 | 62.7 ± 7.1 | 0.123 |
| Left ventricular mass index (g/m^2^) | 97.8 ± 26.5 | 103.4 ± 27.4 | 96.7 ± 26.2 | **<0.001** |
| Left atrial volume index (ml/m^2^) | 41.3 ± 19.4 | 47.7 ± 19.6 | 40.2 ± 19.2 | **<0.001** |
| E (m/s) | 0.6 ± 0.2 | 0.7 ± 0.3 | 0.6 ± 0.2 | **<0.001** |
| A (m/s) | 0.9 ± 0.2 | 1.0 ± 0.3 | 0.9 ± 0.2 | **<0.001** |
| E/e' | 12.6 ± 5.2 | 14.6 ± 6.3 | 12.3 ± 4.9 | **<0.001** |
| Right ventricular systolic pressure (mmHg) | 30.1 ± 11.4 | 34.2 ± 12.8 | 29.4 ± 11.0 | **<0.001** |
| Peak aortic jet velocity (m/s) | 1.7 ± 0.3 | 2.3 ± 0.3 | 1.6 ± 0.2 | **<0.001** |

Values are presented as mean ± standard deviation.

*Abbreviations*: A, late diastolic mitral inflow velocity; AS, aortic stenosis; E, early diastolic mitral inflow velocity; E/e′, early diastolic velocity of the mitral annulus.

Bold formatting indicates statistical significance (p < 0.05).

*p value for progression to AS vs. no progression.
